# Ethanol Diverts Early Neuronal Differentiation Trajectory of Embryonic Stem Cells by Disrupting the Balance of Lineage Specifiers

**DOI:** 10.1371/journal.pone.0063794

**Published:** 2013-05-28

**Authors:** Rosa Sánchez-Alvarez, Saurabh Gayen, Rajanikanth Vadigepalli, Helen Anni

**Affiliations:** 1 Department of Pathology, Anatomy and Cell Biology, Thomas Jefferson University, Philadelphia, Pennsylvania, United States of America; 2 Daniel Baugh Institute for Functional Genomics and Computational Biology, Thomas Jefferson University, Philadelphia, Pennsylvania, United States of America; Baylor College of Medicine, United States of America

## Abstract

**Background:**

Ethanol is a toxin responsible for the neurodevelopmental deficits of Fetal Alcohol Spectrum Disorders (FASD). Recent evidence suggests that ethanol modulates the protein expression of lineage specifier transcription factors Oct4 (Pou5f1) and Sox2 in early stages of mouse embryonic stem (ES) cell differentiation. We hypothesized that ethanol induced an imbalance in the expression of Oct4 and Sox2 in early differentiation, that dysregulated the expression of associated and target genes and signaling molecules and diverted cells from neuroectodermal (NE) formation.

**Methodology/Principal Findings:**

We showed modulation by ethanol of 33 genes during ES cell differentiation, using high throughput microfluidic dynamic array chips measuring 2,304 real time quantitative PCR assays. Based on the overall gene expression dynamics, ethanol drove cells along a differentiation trajectory away from NE fate. These ethanol-induced gene expression changes were observed as early as within 2 days of differentiation, and were independent of cell proliferation or apoptosis. Gene expression changes were correlated with fewer βIII-tubulin positive cells of an immature neural progenitor phenotype, as well as a disrupted actin cytoskeleton were observed. Moreover, Tuba1a and Gapdh housekeeping genes were modulated by ethanol during differentiation and were replaced by a set of ribosomal genes with stable expression.

**Conclusions/Significance:**

These findings provided an ethanol-response gene signature and pointed to the transcriptional dynamics underlying lineage imbalance that may be relevant to FASD phenotype.

## Introduction

Gestational exposure to alcohol can cause developmental abnormalities on the fetus, with up to 1% of all children born in the United States with Fetal Alcohol Syndrome (FAS), the most severe form of Fetal Alcohol Spectrum Disorders (FASD) [1]. Specific craniofacial malformations, prenatal onset of growth deficiency and central nervous system defects are characteristics of FAS [2], which is a leading cause of birth defects and mental retardation. Commonly encountered symptoms are abnormalities of neuronal migration, hydrocephaly, absence of corpus callosum, and cerebellum anomalies [3]. Of the animal models employed for prenatal ethanol exposure (from zebrafish, chicks, guinea pigs, sheep, rodents, to non-human primates), mice have been most useful in defining the vulnerable embryonic stages for teratogenesis [4].

Susceptibility of cells to ethanol during embryogenesis has been addressed in recent years with the use of embryonic stem (ES) cells and their differentiated derivatives. Directed differentiation of human ES cells to neural progenitors, neurons and astrocytes in the presence of ethanol provided insights into the time-course of dysregulation of different neurogenesis-associated genes [5]. In our earlier study, we focused on the early stages of mouse ES cell spontaneous differentiation to embryoid bodies (EBs), corresponding to the period from blastocyst to gastrula, and found that ethanol inhibited asymmetrically the downregulation of Oct4 (also known as Pou5f1), Sox2 and Nanog expression at the protein level [6]. These transcription factors maintain ES cell pluripotency by mutual competition of lineage promoting actions, and in response to intrinsic and extrinsic cues specify the primary germ layers [7]. Therefore, ethanol-induced changes in the level of Oct4, Sox2 and Nanog in EBs indicated potential cell lineage redistribution. In a recent study of retinoic acid (RA)-directed differentiation of ES cells to neuroectoderm (NE) lineage, we demonstrated by flow cytometry-based correlated protein expression in single cells, that ethanol changed in a dose- and time-dependent manner the stoichiometry of Oct4 to Sox2 in distinct cell subpopulations, favoring excess of Oct4 relative to Sox2 [8]. In an elegant work, it was shown that the dosage of Oct4 and Sox2 in early differentiation was critical for lineage specification [9]. Specifically, it was demonstrated that an increased Oct4/Sox2 ratio was responsible for ES cell differentiation to mesoendoderm (ME) lineage, while a higher Sox2/Oct4 ratio promoted NE formation by suppression of the opposing Sox2 or Oct4 signal, respectively. In view of this lineage specifying mechanism of Oct4 and Sox2, our single cell protein data suggested that ethanol misguided cells from NE to ME fate in early stages of differentiation. These transcription factors regulate large number of genes, and ethanol-induced changes in the expression of Oct4 and Sox2 will be therefore amplified at the cellular level and may lead to the neurodevelopmental deficits featured in FASD. Therefore, the motivation of the present study was to uncover the gene signature of the ethanol response and dynamics of gene expression that regulate differentiation trajectories.

Here, we assessed the transcriptional profile of 73 pluripotency, differentiation and signaling genes, including 13 reference gene candidates, during early stages of mouse ES cell differentiation to NE (0, 2, 4, 6 days) in the presence of ethanol (100 mM). The rationale for the choice of differentiation model, ethanol dose and sampling time points was based in our earlier data [8]. Differentiation of ES cells to a single lineage fate facilitated the systematic analysis of regulatory transcription factors and the role of ethanol. Differentiation was driven by RA, an established driver of NE fate, which was employed at a concentration (10 nM), within the physiological *in vivo* range. Under these conditions, an ethanol concentration of 100 mM was found to result in a twofold higher Oct4 protein expression in 3-day differentiated cells [8]. Although lower ethanol doses (25, 50 mM) were found to be efficient in converting the Sox2-Oct4-Nanog positive cells towards the corresponding negative cells, 100 mM ethanol was required for the complete reversal of these subpopulations. Therefore, 100 mM ethanol was employed in the current study. This ethanol concentration mimics binge drinking which has been associated with higher FAS incidence [1]. The progression of differentiation was followed for a period of 0–6 days, which corresponds to E3.5–E9.5 and covers the early germ layer specification processes, and early neurulation. Sampling times were dictated by our earlier findings [8] that showed an ethanol dose-dependent asymmetric modulation of Oct4 and Sox2 expression, as early as after 2 days of exposure, and appearance of fewer neuron-associated Class III β-tubulin isotype (hereafter refer to as βIII-tubulin) immunoreactive cells by 4 days.

Using high-throughput qRT-PCR microfluidic arrays, we identified 33 ethanol-responsive genes with 1.5–20.4 fold (*p*<0.05) modulation of expression. The ethanol-modified transcriptional program was dominated by 19 downregulated pluripotency genes (e.g., Pou5f1, Sox2, Nanog, Klf4, Sall4, Zfp42, Gdf3, and Foxd3) and 14 upregulated differentiation genes (e.g., Cxcl12, Zic1 and Meis1). Additional uncovered ethanol targets involved signaling molecules of the BMP/GDF/FGF4 and STAT3 pathways, known to control fetal development. Importantly, Minimum Spanning Tree-based gene clustering illustrated that ethanol-exposed cells followed a different trajectory than NE during differentiation. Immunocytochemical analysis reconfirmed that fewer ethanol-exposed cells expressed βIII-tubulin of an immature neural progenitor phenotype, and a disorganized actin filaments stress fiber network, linking thus molecular and morphological changes.

## Results

### Ethanol Modifies the Gene Expression Pattern of Transcription Factors and Signaling Molecules Regulating Early ES Cell Differentiation

**Figure 1:**
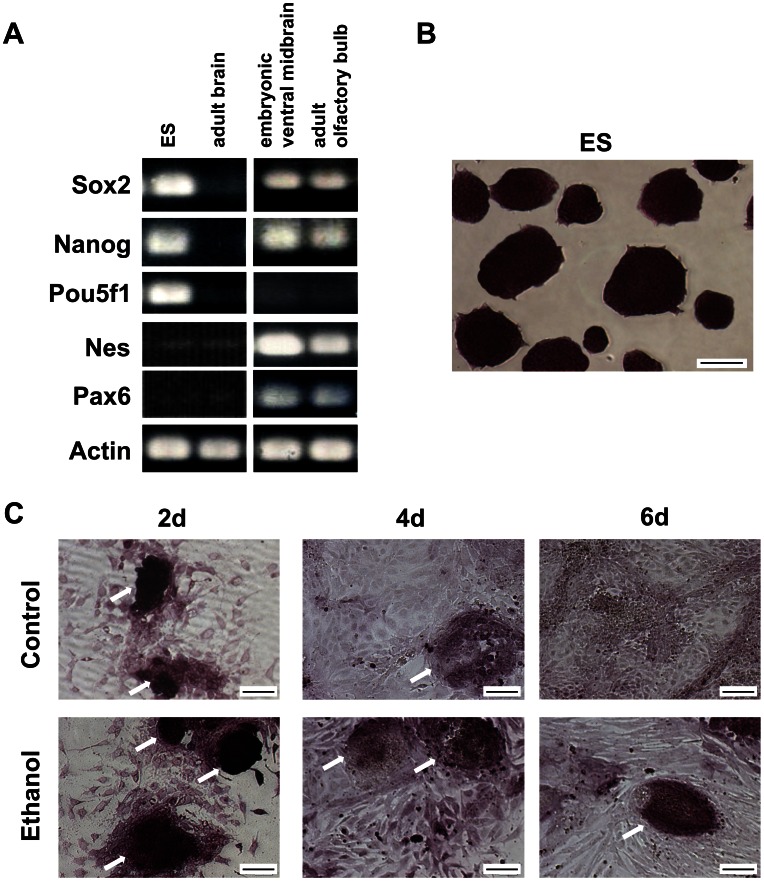
Ethanol changes ES cell morphology during differentiation. (A): ES cells express the core transcription factors (Pou5f1, Sox2, Nanog), but not markers of neural stem cells (Pax6, Nestin), as determined by RT-PCR. Positive controls: mouse embryonic ventral midbrain, adult olfactory bulb; Negative control: adult brain of 4 week-old mice; Loading control: actin. (B): Immunohistochemistry of ES cells for AP shows tightly packed, uniform colonies (deep red color). (C): Staining decreased during differentiation, but more AP-stained colonies and enlarged cells were observed in cells exposed to ethanol, as indicated by arrows. ES cells were differentiated with RA (10 nM) ± ethanol (100 mM) for 2, 4 or 6 days. Scale bar 100 µm.

We carried out multiplex gene expression studies on transcription factors regulating ES cell pluripotency and differentiation, their targets, lineage markers and signaling molecules during NE-directed differentiation of cells exposed to ethanol (100 mM) in a time series (0, 2, 4 and 6 days). ES cells expressed transcripts of the core transcription factors Pouf51, Sox2 and Nanog, but devoid of markers of neural stem cells like Nes (nestin) and Pax6, other than background nestin levels (**Fig. 1A**). Pluripotent ES cells grew in tightly packed colonies with rounded appearance and stained (deep red color) uniformly for alkaline phosphatase (AP), an early marker of undifferentiated cells (**Fig. 1B**). The number of AP-positive colonies and staining intensity progressively decreased during differentiation, and differentiated cells acquired a flattened shape (**Fig. 1C** **,** **upper panel**). However, cells exposed to ethanol were enlarged and residual AP-positive colonies were present (**Fig. 1C** **, lower panel**). Arrows point to the differential ethanol effect on the number and size of AP-stained colonies during differentiation. Overall, morphology analysis indicated that ethanol did not inhibit exit of cells from pluripotency, though the differentiated phenotype was changed.

**Figure 2:**
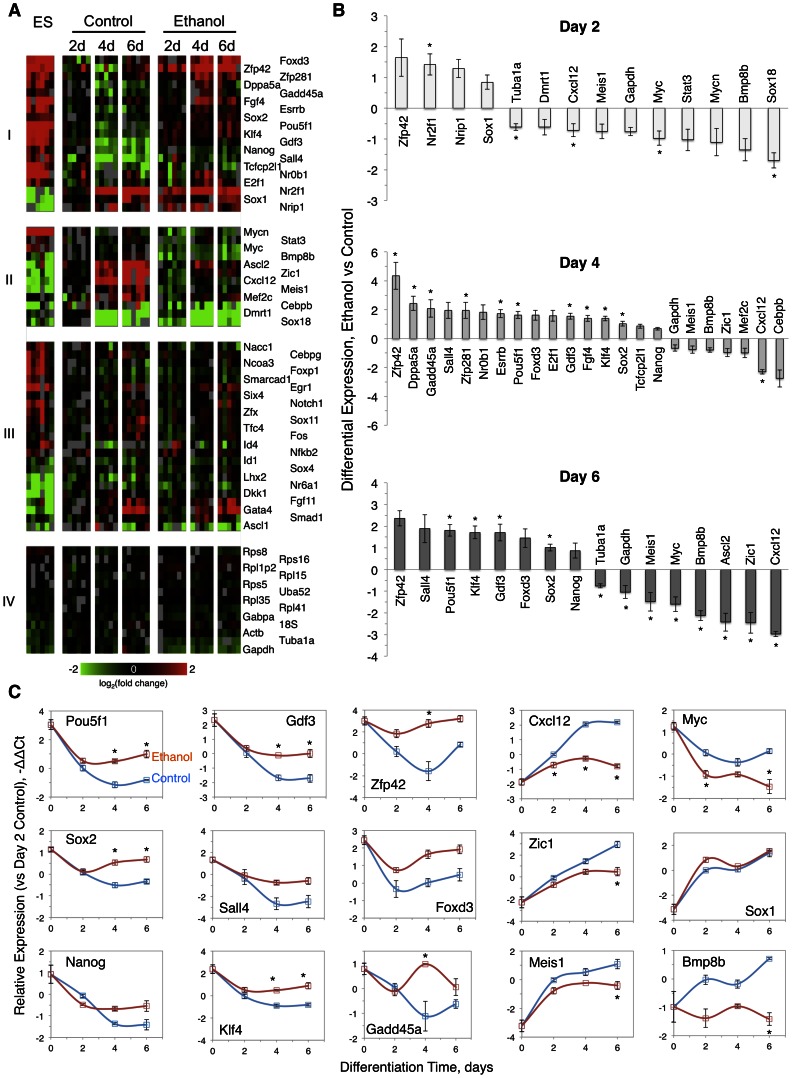
Gene expression ethanol signature during differentiation obtained by multiplex qRT-PCR using microfluidic chips. (A): Clustering of 67 genes of ES and differentiated cells into 4 groups. Ethanol responsive genes in Clusters I (19 upregulated genes) and Cluster II (12 downregulated genes); Ethanol-nonresponsive genes in Cluster III (23 genes) and Cluster IV (13 candidate reference and housekeeping genes). Gene expression fold change (log_2_) in heat maps is presented in color scale. (B): Differentially-expressed genes in response to ethanol exposure during ES cell differentiation for 2 days (14 genes), 4 days (23 genes), and 6 days (16 genes). Gene selection was based on >50% change in expression and p<0.05. Values are average log_2_ fold change ± SEM bars, n = 6 biological replicates. Asterisks indicate statistically significant changes based on adjusted *p* values <0.05. (C): Profile plots of core transcription factors, select major pluripotency-associated transcription factors, core transcription factors targets, proliferation-related genes, signaling molecules, and lineage markers. Gene expression (−ΔΔC_t_) was calculated after reference gene normalization, relative to the median value of day 2 control. Asterisks indicate statistically significant changes with p<0.05 between ethanol and control samples with adjusted *p* values <0.05.

We simultaneously measured the expression of 73 genes across 7 sample groups, and 6 biological replicates using high-throughput qRT-PCR BioMark microfluidic arrays [10]–[11]. The performance of the platform across technical replicates, dilution series and primers is shown in **Figure S1**; gene annotation and primers presented in **Table S1**. The interrogated set was compiled from 60 high-priority genes involved in critical functions in mouse ES cells and their differentiated derivatives [12]–[22], representing mainly transcription factors and their regulators [23], and 13 candidate reference genes. Heat maps in **Figure 2A** depict the expression of 67 select genes with reproducible data across 5–6 biological replicates, distinguishing the ethanol-responsive genes (Cluster I, 19 upregulated genes; Cluster II, 12 downregulated genes) from ethanol-nonresponsive genes (Clusters III–IV). The normalized gene expression profile data are presented in **Table S2.**

Among the 13 candidate reference genes in Cluster IV, Gapdh and Tuba1a were found to be ethanol-regulated (**Figure S2**). The expression of another conventional housekeeping gene Actb, was dependent on differentiation state (**Figure S2, A**). In order to find an appropriate set of reference genes for normalization of gene expression data, 200 candidate reference genes were selected from FunGenES database [12] which was derived from 42 microarrays representing samples obtained at different times of neurogenic differentiation of mouse ES cells. We ranked the top 20 genes according to stability values using geNorm [24] and NormFinder [25] algorithms. Thirteen genes from this list were tested in our sample panel (**Figure S2, B**), and Rpl35, Rps5, Rpl41, Uba52 and Rps16 were chosen as optimal reference genes.

The temporal profile of the ethanol response provided a 33 gene signature (with >50% change in expression, *p*<0.05), and an estimated false positive rate of 11% ((0.05*73)/33), as illustrated in **Figure 2B**. Ethanol modulated the expression of 14 genes early in differentiation (Fig. 2B, top panel), with most genes being decreased by ethanol (10 genes). Of the 23 genes modulated by ethanol during later differentiation, 18 new genes were detected in the window between 2 and 4 days (Fig. 2B, middle panel). The majority of genes in this group had increased expression with ethanol, and the effect was more pronounced than in earlier differentiation (1.6–20.4 fold, 16 genes). Among 16 ethanol-targeted genes on 6 days of differentiation, a late lineage gene Ascl2 was the only new addition (Fig. 2B, lower panel).

**Figure 2C** displays the expression profiles in the course of differentiation of 15 ethanol-upregulated and downregulated genes from various groups. The gene expression of the triad of core transcription factors Pou5f1, Sox2 and Nanog followed the same pattern during differentiation (Fig. 2C, column 1), with an initial decline that leveled off by day 2 in cells exposed to ethanol, in contrast to a further decrease till day 4 in control. The expression of Pou5f1, Sox2 and Nanog was thus elevated in ethanol compared to control on days 4–6 of differentiation (3.3, 2.0 and 1.7 fold, respectively). Gdf3, a gene coregulated with Nanog [26] was elevated 2.9–3.3 fold in cells exposed to ethanol (Fig. 2C, column 2). Gdf3 is a bone morphogenetic protein (BMP) inhibitor that modulates BMP/SMAD signaling [27]. Taken together, the findings on the downregulation of core transcription factors and Gdf3, indicated that ethanol did not prevent the exit of cells from pluripotency, in agreement with morphology analysis (in Fig. 1C). However, higher expression of core transcription factors in cells exposed to ethanol reflected a phenotype resistant to RA differentiation. Importantly, the imbalance in the expression of Pou5f1 relative to Sox2 and Nanog reconfirmed our earlier protein data [8], and pointed out to diversion from NE lineage [9]. Based on the Gdf3 data the SMAD signaling pathway was implicated as an entry point for ethanol action.

Several pluripotency-related genes and targets of core transcription factors had significantly increased expression with ethanol. The expression profile of the zinc finger transcription factors, Klf4 and Sall4, during differentiation was similar to that of core transcription factors, with 3–4 fold higher level on days 4–6 of differentiation in cells exposed to ethanol (Fig. 2C, column 2). Remarkably, ethanol abrogated the downregulation of Zfp42 throughout differentiation, resulting in a 3–20 fold higher expression in cells exposed to ethanol than control (Fig. 2C, column 3). However, the lingering of Zfp42, a pluripotency marker, in cells exposed to ethanol during differentiation, suggested that cells were primed retaining ES cell markers and diminished levels of transcription factors. An important target of core transcription factors, Foxd3 had a bimodal temporal expression pattern which was maintained in cells exposed to ethanol during differentiation, though 3.2 fold elevated (Fig. 2C, column 3). Foxd3 is a transcription factor that suppresses endoderm formation in ES cells [13], while it is a marker of primitive ectoderm (PE) [18]. We interpreted Foxd3 expression profile as indicating that in the presence of ethanol ES cells exit pluripotency and differentiate to PE, though higher Foxd3 expression likely reflected ethanol’s opposition to cell differentiation.

**Figure 5:**
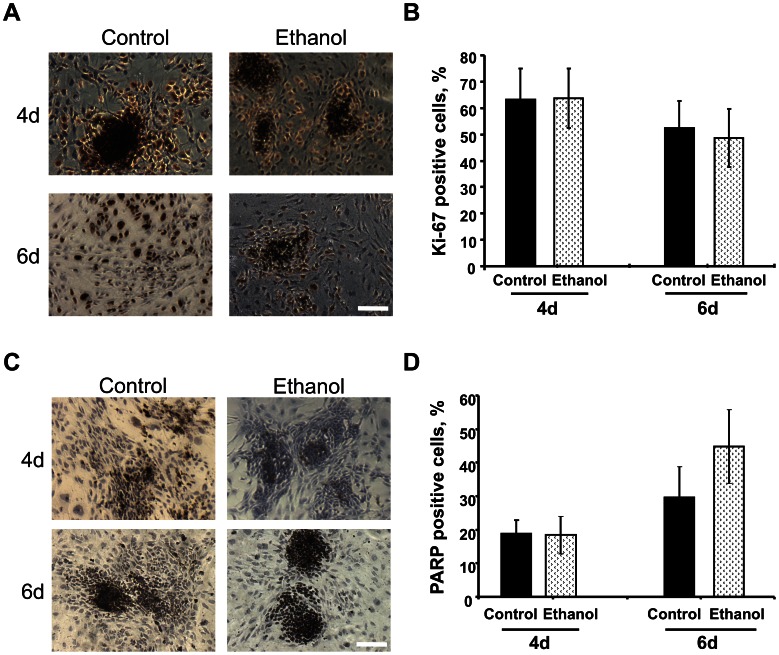
Proliferation and apoptosis were not affected by ethanol during ES cell differentiation. (A–B): Cells were stained with an anti-Ki-67 antibody and nuclei counterstained with hematoxylin (blue color). The percentage of Ki-67 positive proliferating cells (dark brown color) decreased as differentiation progressed from 4 to 6 days, but exposure to ethanol did not affect the proliferation rate. (C–D): Cells were stained with an anti-cleaved PARP antibody and nuclei counterstained with hematoxylin. Number of PARP-positive apoptotic cells (dark brown color) increased during differentiation, but ethanol did not significantly change the rate of apoptosis. A minimum of 15 fields were counted from two separated plates per condition (B, D). Data are expressed as mean ± SEM.

Ethanol inhibited the downregulation during differentiation of several genes that control DNA replication/repair, cell cycle and cell proliferation, such as E2f1, Esrrb, Gadd45a, Sall4, Tcfcp2l1 (Fig. 2A, Cluster I; Fig. 2C, column 3), but accelerated that of Myc and Mycn (Fig. 2A, Cluster II; Fig. 2C, column 5). We note that Essrb, Sall4, and Tcfcp2l1 are major Oct4-interacting proteins [28]. Gadd45a functions in growth arrest and DNA demethylation, and detected first in mouse embryos in the primitive streak and mesoderm at E6.5–E7.5; its expression increased with progression to neurulation [29]. The reciprocal regulation of Gadd45a and Myc observed in cells exposed to ethanol has been established in different cell types [30], and may reflect a stress response. The overall response of cell proliferation-related genes to ethanol prompted us to evaluate cell proliferation and apoptosis induction at the protein level (see below Fig. 5). Like Myc, the level of Stat3 was also decreased by ethanol in early differentiation (Fig. 2A, Cluster II). The onset of ethanol modulation of the expression of Stat3 and its target Myc [31], [32] in early differentiation (2 days) preceded that of core transcription factors (4 days). These findings suggest that ethanol interfered with STAT3 signaling upstream of core transcription factors.

The induced expression of differentiation-related genes (e.g., Cxcl12, Zic1, Mef2c, Meis1, BMP8b, Dmrt1, Sox1) was suppressed by ethanol. There was a strong attenuation (4.9–7.9 fold) by ethanol of Cxcl12 expression on days 4–6 of differentiation (Fig. 2C, column 4). Cxcl12 gene encodes for a chemokine secreted by differentiating ES cells [33], which is important for the development of the nervous system. The significantly diminished steady state level of Cxcl12 with ethanol implied that few cells may advance to NE lineage. In the same vein, the expression of Zic1, a Sox2 target gene [17] which is enriched in neural stem cells [34], increased linearly during differentiation, and decreased 2–5.5 fold by ethanol from day 4 of differentiation onwards (Fig. 2C, Column 4). It is known that Zic1 expression is regulated by BMP/FGF signals [35]. The suppression of Zic1 expression by ethanol is corroborated by a 2.6 fold Fgf4 elevation of transcript detected on day 4 of differentiation (Fig. 2A, Cluster I). Taken together with the ethanol-mediated abrogation of BMP signaling by Gdf3, the Fgf4-Zic4 expression changes reinforce the notion that ethanol brought about defective signaling for NE formation.

In a dual capacity, Mef2c is a transcription factor highly expressed in myocytes and shown to be also an effector of neurogenesis [36]. Data showed a 2 fold decrease of its expression by ethanol (Fig. 2A, Cluster II), which may have implications in ectoderm and mesoderm fate selection. Another pleiotropic transcription factor involved in many developmental processes depending on the cellular context, Meis1 is an early granule cell progenitor marker [34], that was inhibited by ethanol, remaining 1.7–2.8 fold lower than control in later differentiation stages (Fig. 2C, Column 4). Furthermore, ethanol interfered with the expression of germ cell-specific genes BMP8b and Dmrt1. Ethanol abrogated BMP8b upregulation in differentiating ES cells (Fig. 2C, column 5). BMPs, including BMP8b are secreted from the extraembryonic ectoderm starting on E6 in mice and induce activation of transcription factors via SMAD1/5 in primordial germ cells of the extraembryonic mesoderm appearing on E7.25 [37]. This finding manifested that ethanol disrupted proper signaling at the ectoderm/mesoderm interface. Likewise, Dmrt1 was found to be downregulated by ethanol (Fig. 2A, Cluster II). Dmrt1 is a transcription factor able to convert mouse fibroblasts into embryonic Sertoli-like cells, when combined with Nr5a1, Wt1, Gata4 and Sox9 [38]. Overall, our data on differentiation-related genes pointed out to a reduced potential of cells exposed to ethanol to proper differentiation into neuronal progenitors.

Consistent with the role of Sox2 in neural differentiation, other Sox genes were also found to be modulated by ethanol. The gene expression of Sox1 sharply increased early in differentiation and was upregulated transiently 1.8 fold on day 2 of differentiation in cells exposed to ethanol (Fig. 2C, Column 5). Sox1 is a marker of PE, and as the regulation of Foxd3 indicated, ethanol promoted switching of ES cells into PE lineage. The elevated Sox1–Sox2 expression during differentiation of cells exposed to ethanol likely disturbed the cooperation with other transcription factors required for coordinated lineage selection and progression to neurogenesis [39].

### Ethanol Inhibits with Formation of Neuronal Cells and Disorganizes the Actin Filaments Network

**Figure 3:**
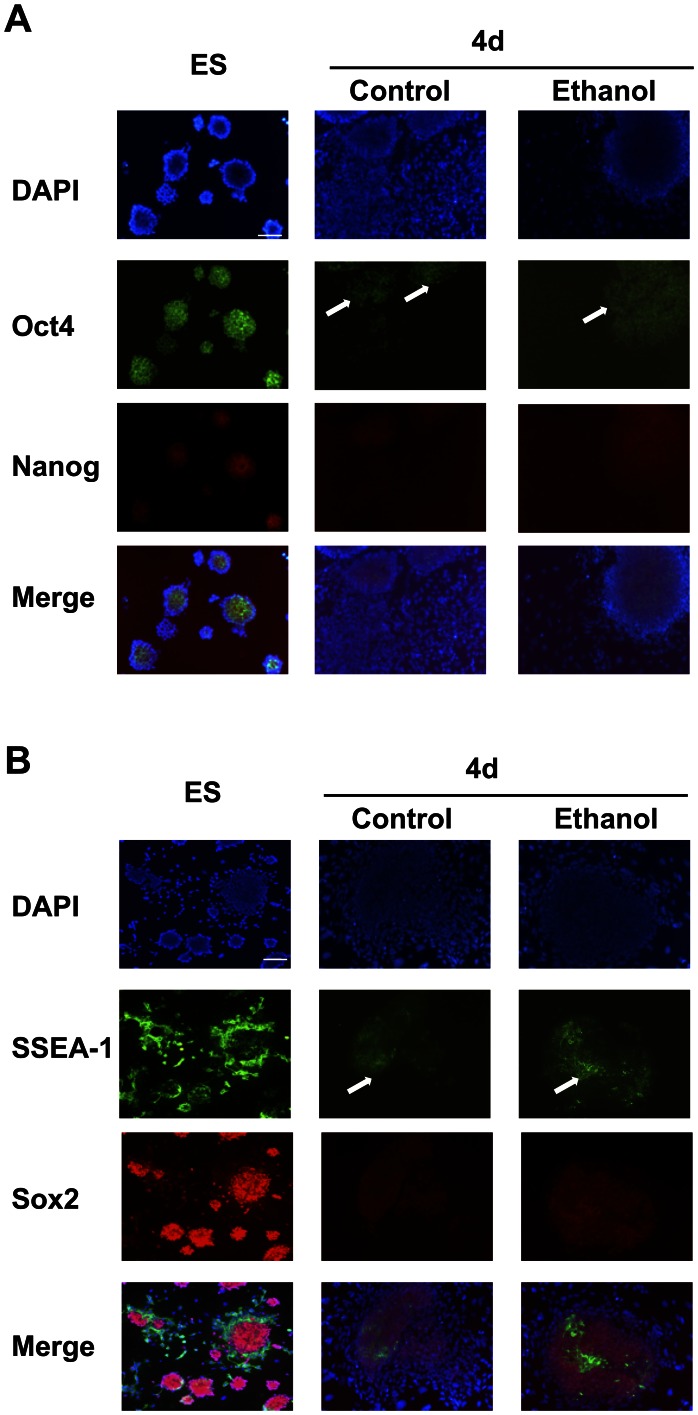
Ethanol inhibited the downregulation of core transcription factors Oct4, Sox2, Nanog, and pluripotency marker SSEA-1 in 4-day differentiated cells. (A–B): Fixed cells were stained with antibodies labeled with either Alexa Fluor 488 (Oct4, SSEA-1) or Alexa Fluor 546 (Sox2, Nanog), and nuclei were visualized with DAPI. Merged images showed nuclear localization of core transcription factors Oct4, Sox2 and Nanog, and SSEA-1 on the cell membrane. Arrows indicate decreased expression of these proteins during differentiation, but higher expression in ethanol-exposed cells. Representative photomicrographs from n = 3. Scale bar 100 µm.

We assessed the consequences of ethanol-mediated aberrant gene regulation on neuronal differentiation through *in situ* protein expression (**Fig. 3**). The expression of core transcription factors Oct4, Sox2 and Nanog was restricted to ES cell colonies, and markedly downregulated as differentiation proceeded for 4 days and the number of colonies decreased, becoming undetectable after 6 days of differentiation (data not shown). More residual core transcription factor staining was observed in cells exposed to ethanol compared to control, as indicated by arrows. The expression of the classical surface pluripotency marker stage-specific embryonic antigen-1 (SSEA-1, also known as Lewis X or CD15) carbohydrate antigenic epitope [40] was similarly retained in cells exposed to ethanol (see arrows). Overall, our *in situ* protein data reconfirmed the ethanol-mediated changes of core transcription factors at the transcript level. Moreover, cell aggregates present during differentiation in cells exposed to ethanol were attributable to undifferentiated colonies expressing core transcription factors and pluripotency markers AP (in Fig. 1C) and SSEA-1.

**Figure 4:**
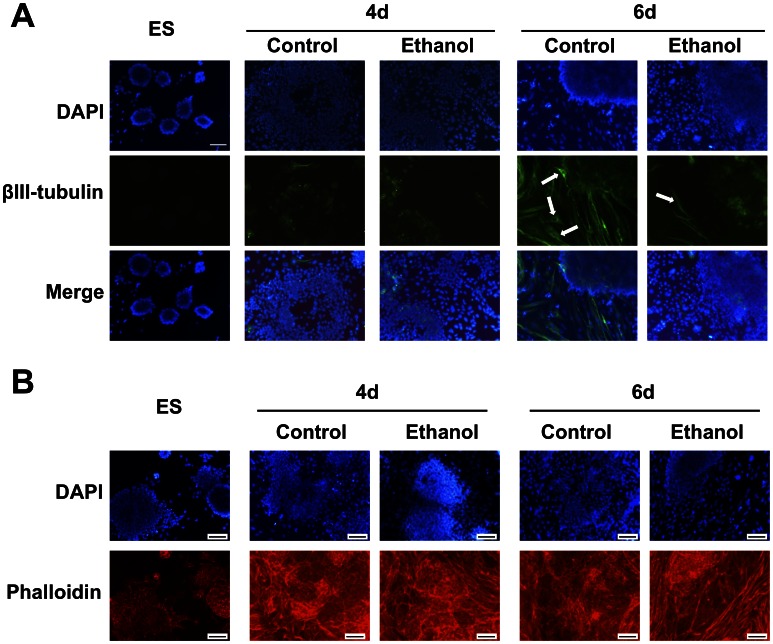
Ethanol decreased the formation of early neural progenitors and caused disorganization of the cytoskeleton during differentiation. (A): Staining of neuronal βIII-tubulin using an Alexa Fluor 488 labeled monoclonal antibody showed appearance of early neural progenitors at 4 days of differentiation. Nuclei were visualized with DAPI. The number of neural progenitors increased significantly at later stages of differentiation. Ethanol exposure markedly decreased the overall number of βIII-tubulin-immunoreactive cells, as highlighted by arrows in comparison to control (B): Staining of actin filaments with rhodamine-conjugated phalloidin. Nuclei were visualized with DAPI. The structural arrangement and cellular organization of the actin filament network was modified during differentiation of cells exposed to ethanol, producing an elongated pattern in the cytoskeleton stress-fibers. (A-B): Representative photomicrographs from n = 3. Scale bar 100 µm.

Early neural progenitors were detected by staining for βIII-tubulin at 4 days of differentiation, and significantly increased at 6 days (**Fig. 4A**). Ethanol exposure reduced markedly the staining and number of βIII-tubulin-positive cells. In early differentiation, these cells had mostly rim-like staining of the perinuclear cytoplasm, and few (<5%) stained at the proximal end of the developing neurites. In later differentiation, cells with small cell bodies and longer projections also appeared. However, in the presence of ethanol, most βIII-tubulin-positive cells had an immature phenotype, and fewer differentiated cells expressing Tuj1 with short processes were observed at 6 days in the presence of ethanol compared with control.

Since βIII-tubulin is an important component of the cytoskeleton necessary for the correct guidance and migration of neural progenitors [41], and ethanol decreased its expression in differentiated cells, we evaluated the effect of ethanol on the expression, and organization of actin filaments. **Figure 4B** shows that ethanol did not modify actin expression in phalloidin-stained cells. However, the structural arrangement and cellular organization of the actin filament network during later differentiation stages was modified with appearance of an elongated pattern in the cytoskeleton stress fibers. Disruption of cytoskeletal microtubules and microfilaments by ethanol has been reported in several cell types, such as human ES cell-derived neural progenitors [5], mouse neural crest cells [42], human neuroblastoma LA-N-5 cells [43], mouse dorsal hippocampus-derived CA1 pyramidal neurons from fetuses exposed to ethanol on gestational day (GD) 15 [43], and rat hippocampus neurons exposed to ethanol on GD16 [44]. Moreover, such cytoskeletal reorganization upon ethanol exposure has been detected *in vivo* in mice and linked to altered signaling pathways [45]. These cytoskeletal changes may be related to the elongated shape of differentiated cells in the presence of ethanol (seen in Fig. 1C), and potentially correlated to ES cell differentiation away from NE lineage.

### Cell Poliferation and Apoptosis during ES Cell Differentiation are Unaffected by Ethanol

In view of ethanol-dependent changes in the expression of several cell proliferation genes during differentiation (seen in Fig. 2), we examined the tightly coordinated processes of cell proliferation and apoptosis. Immunocytochemical staining of fixed cells for nuclear antigen Ki-67, showed that cells whether differentiated with or without exposure to ethanol were highly proliferative (dark brown nuclei), especially in areas of cell aggregates, and proliferation decreased as a function of differentiation time (**Fig. 5** **A–B**). These findings are in agreement with earlier flow cytometry measurements of incorporation of a uridine analog, where the percentage of 4-day differentiated cells in S phase was unchanged in ethanol (54.4%) compared to control (52.4%), and decreased in comparison to ES cells (76.6%) [8]. Thus, ethanol did not inhibit overall the cell cycle during ES cell differentiation. It is likely that the observed proliferation-related gene expression changes with ethanol exposure were part of a compensatory response. A recent study has similarly demonstrated that ethanol did not affect the rate of proliferation of human ES cell-derived neural progenitors [5].

There is an unequivocal link between apoptosis and differentiation [46], and lineage selection [47]–[49]. We therefore looked into the contribution of ethanol to apoptosis using cleaved poly ADP-ribose polymerase (PARP) as a marker. Immunocytochemical analysis clearly demonstrated that the number of PARP-positive cells (dark brown nuclei) in fixed cells increased as a function of differentiation time, but was not dependent on exposure of cells to ethanol (**Fig. 5** **C–D**). Ethanol-induced apoptotic signals reported in other differentiation platforms were not detectable in our β-mercaptoethanol protected from oxidative stress culture environment. We reported earlier that apoptosis measured by Annexin V-propidium iodide increased 6 fold upon ES cell differentiation, but differences were not detected between control and ethanol-exposed differentiated cells after 4 days of differentiation [8]. Removal of β-mercaptoethanol from differentiation media increased significantly apoptosis [50] that was synergized by ethanol [unpublished data]. By the same token, ethanol increased apoptosis during EB differentiation in antioxidant-free environment [6].

## Discussion

Transcriptional regulation is central to pluripotency and differentiation of ES cells. It is known that the core transcription factors Oct4, Sox2 and Nanog in ES cells interact combinatorially to regulate gene expression [51]. We have previously studied the interference of ethanol with this transcriptional network during early differentiation of mouse ES cells [6], [8]. Our findings demonstrated that ethanol delayed to different extent the decline of Oct4, Sox2 and Nanog protein level in two differentiation systems; spontaneously formed EBs, representing the three primary germ layers [6], and RA-induced differentiated NE cells [8]. Moreover, an excess of Oct4 relative to Sox2 in ethanol-exposed cells suggested induction of a divergent ME cell fate in mouse ES cells under RA-directed differentiation conditions. We therefore investigated here how the imbalance of Oct4 and Sox2 in ethanol-exposed cells differentiated towards NE fate with RA, affected their differentiation trajectory.

### Ethanol Gene Signature of Early Cell Differentiation

Out of 73 key genes measured by multiplex qRT-PCR, the expression of 33 genes was altered by ethanol, in at least one or more differentiation times (Figs. 2A, 2B). Cells exposed to ethanol during differentiation were capable of initially downregulating the gene expression of Pou5f1, Sox2 and Nanog, albeit in an asymmetric manner, while maintaining a significantly higher transcript level than control (Fig. 2C). These results were consistent with changes at the *in situ* protein level (Fig. 3), as well as with earlier flow cytometry-based measurements [8]. We interpret these changes as an apparent resistance of ethanol-exposed cells to RA-directed differentiation. This notion was reinforced by the elevated expression of pluripotency markers AP (Fig. 1C) and SSEA-1 (Fig. 3). The protein expression of SSEA-1 was also significantly higher in ethanol-exposed EBs [6]. Overall, cells exposed to ethanol during differentiation presented a phenotype similar to ES cells overexpressing Nanog [13].

#### Targets of core transcription factors

The gene expression of several targets of core transcription factors was higher than control during differentiation of ethanol-exposed cells. The list included Klf4, Dppa5a, Nr0b1 (targeted by Oct4); Esrrb, Sall4, Zfp42, Gdf3, Fgf4 (targeted by Oct4, Nanog) and Foxd3 (targeted by Oct4, Sox2) (Figs. 2B, 2C). Targets were tentatively identified based on response upon suppression of individual core transcription factors in ES cells [17].

Klf4 is a zinc finger transcription factor and major pluripotency gene that in a cocktail with Oct4, Sox2 and Myc was able to reprogram mouse embryonic fibroblasts into induced pluripotent stem (iPS) cells [52]. It can be replaced by Esrrb, an interacting partner of Oct4 that regulates the expression of Nanog [28], in converting mouse embryonic fibroblasts into iPS cells in combination with Oct4 and Sox2 [53]. Sall4 is also a zinc finger transcription factor, which associates with Oct4 and Nanog [28], [54], and stabilizes the undifferentiated ES cell state [55]. Increased expression of Klf4, Esrrb and Sall4 in ethanol-exposed cells reflected a phenotype resistant to differentiation.

In contrast to the trend observed with other core transcription factors targets, Zfp42 was not downregulated, and Foxd3 had a bimodal pattern during differentiation of ethanol-exposed cells. Foxd3 encodes a transcriptional suppressor of differentiation, which is important for the maintenance of the inner cell mass, ES and epiblast cells. Overexpression of Zfp42 or Foxd3 (via Nanog) was reported to attenuate RA-induced ES cell differentiation [13], [56].

Several Nanog-interacting proteins, like Gdf3, Nr0b1 and Zfp281 [54] had elevated transcripts in ethanol-exposed cells during differentiation. Higher Gdf3 expression in ethanol-exposed cells was correlated with Nanog overexpression, since both genes are in a cluster regulated by Oct4 [26]. Gdf3 is a ligand of the transforming growth factor-beta (TGFβ) family, classified in the BMP/growth differentiation factor (GDF) branch that functions as a BMP inhibitor [27]. Therefore, ethanol-exposed cells with an increased Gdf3 likely have aberrant BMP/SMAD signaling. A disruption of the TGFβ pathway was recently reported in a focused transcriptomic study of human neural stem cells treated with ethanol [57]. *In vivo*, Gdf3 is present within the inner cell mass of the blastocyst and the ectoderm during mouse pregastrulation stage, where it establishes a BMP gradient essential for the formation of primitive streak [58]. Importantly, Gdf3 mutations are associated with Klippel-Feil syndrome of skeletal abnormalities and alcohol drinking during pregnacy (http://ghr.nlm.nih.gov/condition/klippel-feil-syndrome).

#### Differentiation genes

Ethanol inhibited the expression of several differentiation-induced genes, such as Zic1, Cxcl12, Meis1, Mef2c and Sox1 (Figs. 2B–C). Zic1 is a transcription factor of the Zic family [59] that potentiates (with Ascl1, Pou3f2, Myt1l, Pou3f4, Olig2) the conversion of mouse fibroblasts to neurons [60].

The Zic1 transcript was first detected at E7.0 mice preferentially in prospective NE cells [61]. The decreased Zic1 expression by ethanol may result from defective BMP (via Gdf3) and Fgf4 signaling. Additional disrupted signaling to NE by ethanol was indicated by the limited expression of Cxcl12 transcripts of α, β, γ isoforms (detected by our primer) [62]. In embryogenesis CXCL12 and its unique receptor CXCR4 are expressed during gastrulation in the E7.5 ectoderm/mesoderm border to guide appropriate cell migration and the development of the nervous system [63].

Meis1 protein is expressed in interaction with other transcription factors in several organs during mouse embryogenesis. At the gastrulation stage, Meis is expressed in the primitive streak (as is the case for Gdf3). Meis has been identified recently as a mesodermal gene and target of Brachyury in EBs [64]. Finally, Mef2c is a transcription factor capable of converting precursor cells into myocytes, but also shown to promote formation of neuronal progenitor cells [36]. Taken together, the inadequate expression of NE differentiation-related genes in ethanol-exposed cells indicated that fewer NE cells were likely formed. Importantly, immunocytochemical staining for early neuronal marker βIII-tubulin established that there were fewer immature neuronal cells under ethanol-exposed conditions (Fig. 4A).

### Ethanol-induced Cell Lineage Divergent Trajectory in Early Differentiation

**Figure 6:**
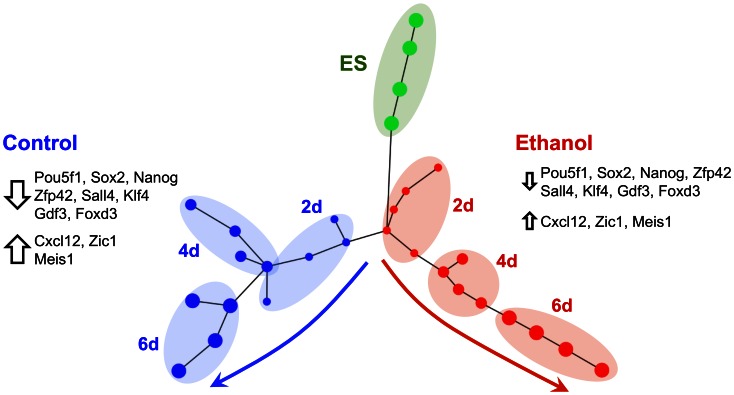
Diversion of ES cell differentiation in the presence of ethanol away from NE lineage. Clustered gene expression data are presented along a Minimum Spanning Tree. Nodes represented biological samples, and time point labels indicated differentiation day. The gene expression dynamics in ethanol-exposed cells *(red)* suggested that differentiation of ES cells *(green)* was not delayed but rather driven away from neuroectodermal fate *(blue)*. Important genes with pronounced differential expression in ethanol-exposed cells are highlighted. Arrows indicate up- or down-regulation and relative size in ethanol compared to control cells.

We visualized the progression of the gene expression dynamics through ES cell differentiation (Fig. 2), using a Minimum Spanning Tree based clustering approach (**Fig. 6**). In this scheme all samples were included (i.e., ‘spanning’) in a connected single-linkage dendrogram (‘i.e., ‘tree’), while total dissimilarity was minimized (i.e., ‘minimum’). As a result, samples closely related are connected and reveal hierarchical relationships as evidence of non-random structure. Accordingly, the overall gene expression profile of cells exposed to ethanol for 2 days of differentiation is closer to ES cells than control. However, ethanol-exposed cells followed a different trajectory than control during later stages of differentiation, days 4 to 6. These trajectories demonstrated that cells exposed to ethanol were not merely ‘falling behind’ in terms of differentiation, but rather ethanol led to an altered transcriptional program and a system state that is not entirely intermediate to ES and RA-directed NE differentiation. Earlier work at single cell level has shown that aberrant expression of Oct4 relative to Sox2 in ethanol-exposed cells is critical to the selection of lineage fate [8]. Additional ethanol entry points uncovered in this study involve signaling via the BMP/GDF/FGF4 and STAT3 pathways which control fetal development.

In conclusion, cells exposed to ethanol in early stages of differentiation exhibited a modified gene expression pattern. Ethanol caused a 3–20 fold differential expression of core transcription factors and several genes belonging to the groups of major pluripotency genes, cell lineage markers, proliferation genes, and signaling molecules (cited in increasing order of differential expression: Esrrb, Klf4, Gdf3, Sox18, Myc, E2f1, Nr2f1, Fgf4, Pou5f1, Nr0b1, Zfp281, Sall4, Gadd45a, Bmpb8a, Ascl2, Dppa5a, Zic1, Cebpb, Cxcl12 and Zfp42). The gene expression dynamics of cells exposed to ethanol uncovered a derailing of the RA-directed NE fate in early differentiation. An ethanol-induced divergence of cell fate to ME was supported by the asymmetric change in the transcriptional and protein expression of the core transcription factors Oct4 and Sox2, which favored an excess of Oct4. Several Oct4 and Sox2 targets affected by ethanol exposure were identified in our screening that reconfirmed an altered transcriptional network. Moreover, BMP/GDF/FGF4 and STAT3 signaling pathways were disrupted, indicating ethanol entry points into the transcriptional network.

## Methods

### Maintenance and Differentiation of ES Cells

Mouse ES cells (E14Tg2A) were cultured in adherent monolayer, as described previously [8]. To induce ES cell differentiation, cells were plated at low density, and medium was supplemented with10 nM all-*trans* RA 24 hours after seeding in ES cell medium, according to [8]. Ethanol (100 mM) was added to culture medium at the onset of differentiation for 2, 4 or 6 days. Ethanol concentration and differentiation sampling times were selected based on earlier dose-response and time-course studies [8].

### Alkaline Phosphatase Staining

Cells were stained with alkaline phosphatase (AP) as per manufacturer’s protocol (Millipore, Billerica, MA). Images of cells were obtained with a bright field Olympus IX2-SL microscope equipped with a Q color 3 digital camera and processed with cellSens program.

### Immunocytochemistry

Cells were fixed and permeabilized with standard techniques. The primary antibodies used were: mouse monoclonal anti-SSEA-1 (sc-21702, 1∶250), mouse monoclonal anti-Oct3/4 (sc-5279, 1∶250), rabbit polyclonal anti-Nanog (sc-33760, 1∶250), and goat polyclonal anti-Pax6 (1∶50), all from Santa Cruz Biotechnology (Dallas, TX), and mouse monoclonal anti-βIII-tubulin (Tuj1) (ab7751, 1∶200) from Abcam (Cambridge, MA). To visualize actin filaments cells were incubated with rhodamine-conjugated phalloidin (Invitrogen, 1∶500). Conjugated secondary antibodies were: Alexa Fluor-488 chicken anti-mouse, Alexa Fluor-546 donkey anti-goat, Alexa Fluor-546 goat anti-rabbit (1∶250; Invitrogen). Cells were mounted with ﬂuorescent mounting medium and DAPI to visualize nuclei (Vectashield, Vector Laboratories, Burlingame, CA). Fluorescence photomicrographs were acquired with a CKX41 digital video camera connected to an Olympus inverted fluorescent microscope.

### Cell Proliferation and Apoptosis

Fixed cells were incubated with rabbit polyclonal anti-Ki-67 (1∶50) or rabbit monoclonal anti-cleaved poly (ADP-ribose) polymerase (PARP) (1∶250) (Abcam) and stained as per avidin-biotin immunoperoxidase kit instructions (ABC, Vector Laboratories). Nuclei were counterstained with hematoxylin. Each experiment was carried out in two plates per condition, and at least 15 fields were counted per plate. Images were processed with ImageJ (http://rsb.info.-nih.gov/ij).

### RNA Extraction, cDNA Synthesis and RT-PCR

Total RNA was isolated with TRIzol reagent (Invitrogen), and purified with DNA-free RNA kit (Zymo Research, Irvine, CA) before concentration and integrity were assessed. RNA was reverse transcribed into cDNA using random hexamers and SuperScript III, following manufacturer’s instructions (Invitrogen). For the analysis of select genes PCR reactions were carried out in a thermal cycler (MI Research), using gene-specific primers for Oct4, Sox2, Nanog, Nestin and Pax6 (Roche, Indianapolis, IN), and Green Hot Start Master Mix Polymerase (Promega, Madison, WI). Roche’s Universal Probe Library Assay Design Center (www.universalprobelibrary.com) was used to design intron spanning PCR primers and probes. cDNA samples were selectively preamplified for 14 cycles [10]. Gene expression data were obtained using Fluidigm’s high-throughput qRT-PCR BioMark microfluidic arrays (http://www.fluidigm.com; South San Francisco, CA) as described earlier [11]. Each dynamic 48.48 dynamic array chip measures in parallel 2,304 assays (48 assays in 48 samples).

### Selection of Reference Genes

The expression of conventional housekeeping genes Gapdh, Tuba1a and Actb, was dependent on differentiation state and ethanol treatment (Figure S1, A). A total of 200 candidate reference genes with stable expression (signal intensity ≥2^8^, coefficient of variance ≤0.6%) were selected from AVEF-1 dataset [12] for gene expression normalization. After stability ranking of the top 20 genes by both geNorm [24] and NormFinder [25] algorithms, 13 genes were tested in our sample panel (Figure S1, B), and Rpl35, Rps5, Rpl41, Uba52 and Rps16 were chosen as optimal reference genes. The mean expression value of these 5 genes per experimental condition was used to normalize the gene expression data. The average of the cycle threshold (C_t_) and −ΔΔC_t_ values were calculated according to [65].

### Statistical Analysis

Data analysis was performed in R program (http://www.r-project.org/). We performed initially Student’s t-tests based-comparisons of gene expression data, and two-way ANOVA as appropriate in each statistical comparison (‘aov’ function in R), followed by statistical significance testing of relevant comparisons using Tukey’s ‘Honest Significant Difference’ method based on the Studentized range statistics at a family-wise confidence interval of 95% (‘TukeyHSD’ function in R). A *p* or post-hoc adjusted *p* value <0.05 was considered statistically significant. The estimated false positive rate was 11%. Data were obtained from n = 6 biological replicates, and n = 4 BioMark chips.

### Minimum Spanning Tree Visualization

Gene expression data were visualized as Minimum Spanning Tree, based on Pearson correlation, in an approach similar to other systems [66], [67]. Nodes in the spanning tree represented biological samples. Calculations were carried out using the *spantree* function in the *vegan* library in the R platform for statistical analysis (http://www.r-project.org).

## Supporting Information

Figure S1 **Performance of the 48x48 high-throughput qRT-PCR microfluidic array among biological and technical replicates and different set of primers for various assays.** (A): Comparison of C_t_ curves between 2 technical replicates (pre-amplified samples) for 3 assays of Sox2, Myc and Actin in ES cells showed similar amplification curves. (B): A typical 6-point standard curve of a primer pair for Nanog using a dilution series of mouse DNA as a template. Calibration curve was constructed with 10x dilutions over five orders of magnitude. (C): Range of ΔC_t_ values of primers for the core transcription factors Pou5f1, Sox2 and Nanog across 5 samples (ES cells, Control Day 2, Ethanol Day 2, Control Day 4, Ethanol Day 4).
(TIF)

Figure S2 **Selection of optimal reference genes.** (A): Profile plots of Gapdh, Tuba1a and Actb show that expression of conventional housekeeping genes depends on differentiation and/or ethanol exposure. Gene expression (−ΔΔC_t_) was calculated after reference gene normalization, relative to the median value of 2 day control. Asterisks indicate statistically significant changes with p<0.05 between ethanol and control or different time points. (B): Expression stability of 13 candidate reference genes across experimental conditions was calculated using the GeNorm and NormFinder algorithms. The top 5 common genes with lowest stability (low variability) are highlighted. The mean expression value of these genes per experimental condition was used to normalize the gene expression data.
(TIF)

Table S1 **List of primers and probes used in qRT-PCR.**
(XLS)

Table S2 **Normalized gene expression values used for the construction of the heatmap in** **Figure 2A** **.** NA indicates missing data from failed assays.
(XLS)
