# Supplementary figures and images for: Ethanol Diverts Early Neuronal Differentiation Trajectory of Embryonic Stem Cells by Disrupting the Balance of Lineage Specifiers
Source: PLoS One. 2013 May 28;8(5):e63794. doi: 10.1371/journal.pone.0063794 (PMC3665827; doi:10.1371/journal.pone.0063794)

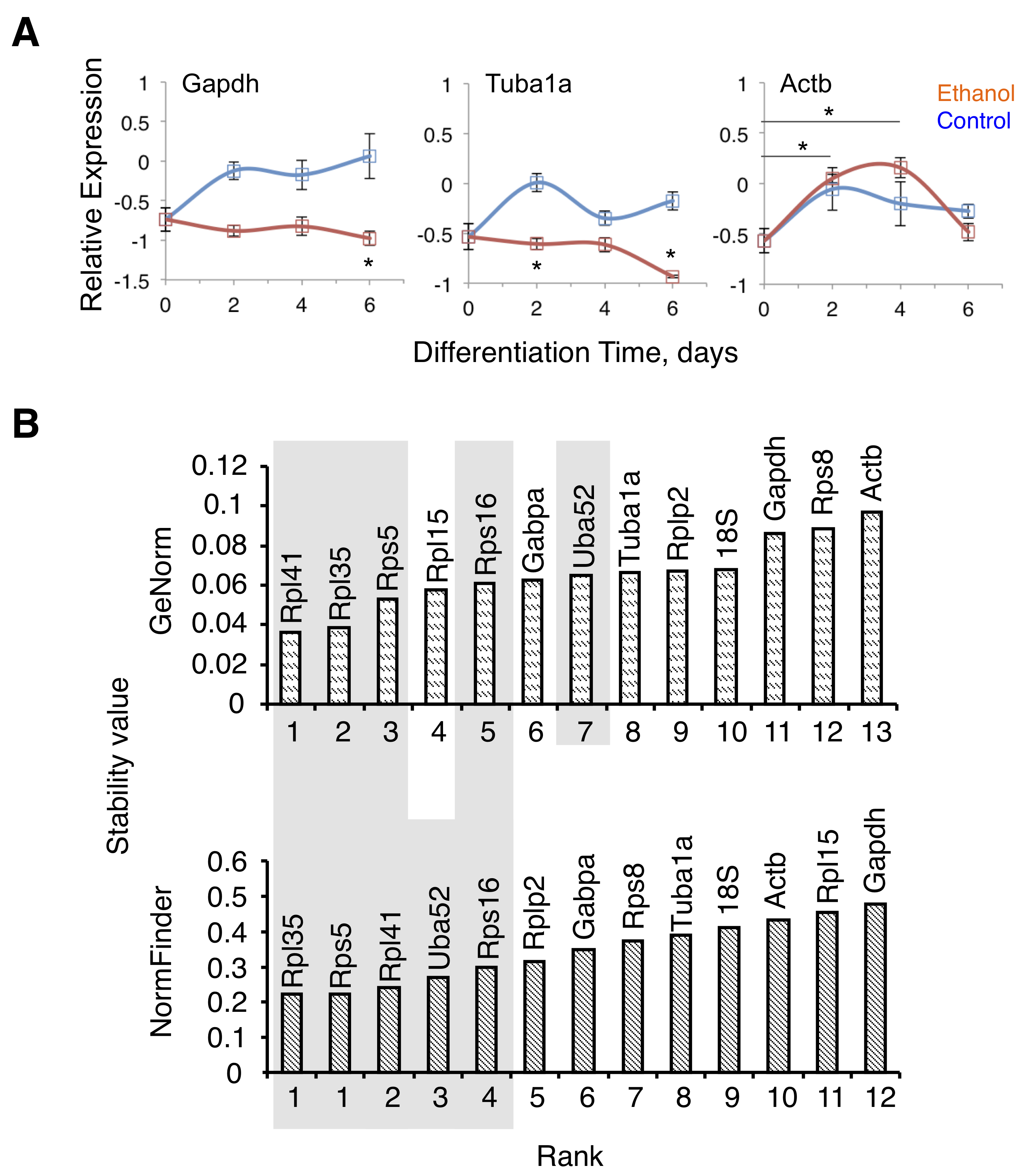

Supplement: Figure S1 — Performance of the 48x48 high-throughput qRT-PCR microfluidic array among biological and technical replicates and different set of primers for various assays. (A): Comparison of Ct curves between 2 technical replicates (pre-amplified samples) for 3 assays of Sox2, Myc and Actin in ES cells showed similar amplification curves. (B): A typical 6-point standard curve of a primer pair for Nanog using a dilution series of mouse DNA as a template. Calibration curve was constructed with 10x dilutions over five orders of magnitude. (C): Range of ΔCt values of primers for the core transcription factors Pou5f1, Sox2 and Nanog across 5 samples (ES cells, Control Day 2, Ethanol Day 2, Control Day 4, Ethanol Day 4). (TIF) [file pone.0063794.s001.tif]

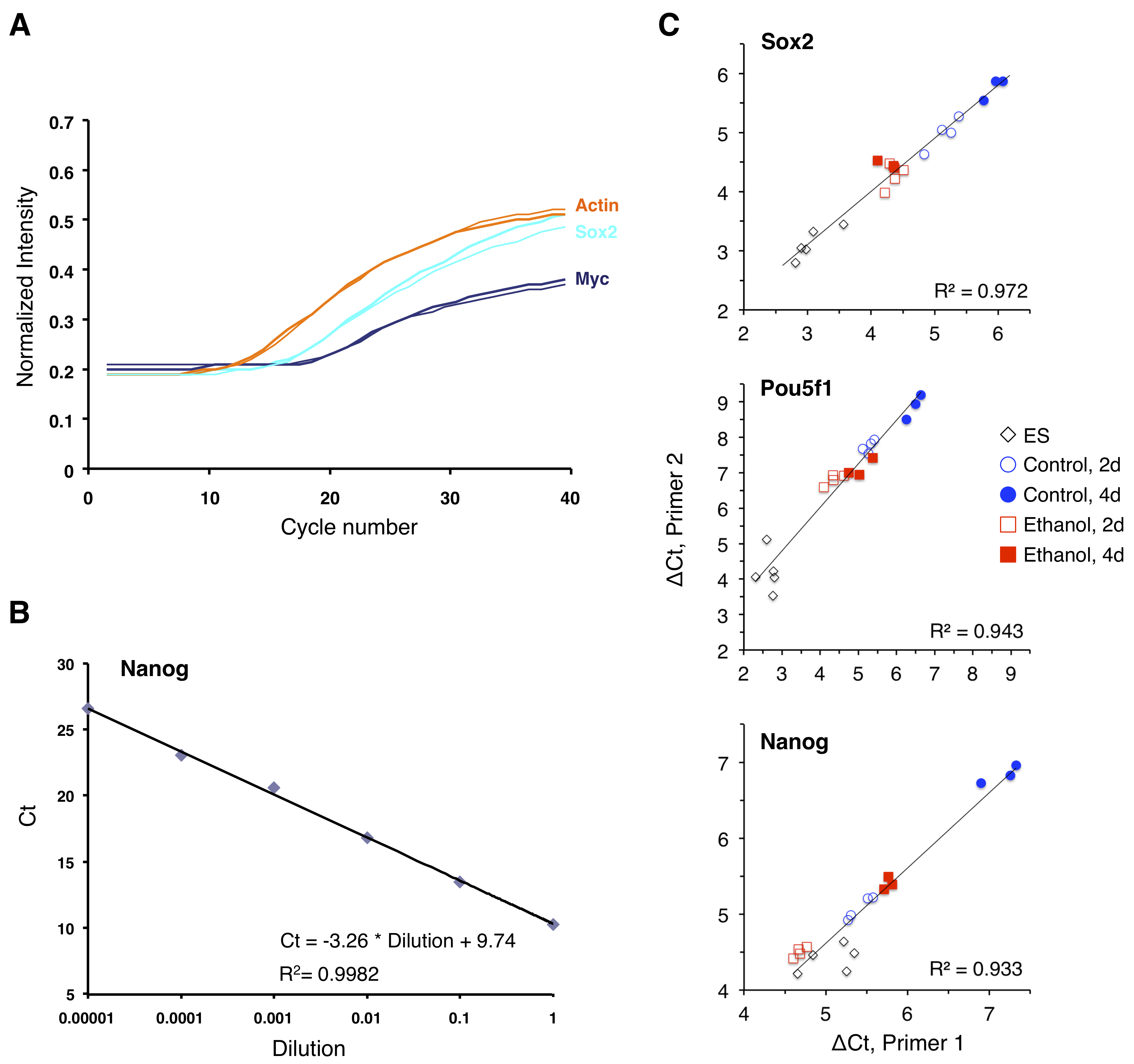

Supplement: Figure S2 — Selection of optimal reference genes. (A): Profile plots of Gapdh, Tuba1a and Actb show that expression of conventional housekeeping genes depends on differentiation and/or ethanol exposure. Gene expression (−ΔΔCt) was calculated after reference gene normalization, relative to the median value of 2 day control. Asterisks indicate statistically significant changes with p<0.05 between ethanol and control or different time points. (B): Expression stability of 13 candidate reference genes across experimental conditions was calculated using the GeNorm and NormFinder algorithms. The top 5 common genes with lowest stability (low variability) are highlighted. The mean expression value of these genes per experimental condition was used to normalize the gene expression data. (TIF) [file pone.0063794.s002.tif]
